# Chronologically scheduled snacking with high-protein products within the habitual diet in type-2 diabetes patients leads to a fat mass loss: a longitudinal study

**DOI:** 10.1186/1475-2891-10-74

**Published:** 2011-07-14

**Authors:** Santiago Navas-Carretero, Itziar Abete, M Angeles Zulet, J Alfredo Martínez

**Affiliations:** 1 Department of Nutrition, Food Science, Physiology and Toxicology, University of Navarra, Irunlarrea 1, 31008 Pamplona, Spain

## Abstract

**Background:**

Obesity is the most relevant overnutrition disease worldwide and is associated to different metabolic disorders such as insulin resistance and type-2 diabetes. Low glycemic load foods and diets and moderately high protein intake have been shown to reduce body weight and fat mass, exerting also beneficial effects on LDL-cholesterol, triglyceride concentrations, postprandial glucose curve and HDL-cholesterol levels. The present study aimed at studying the potential functionality of a series of low glycemic index products with moderately high protein content, as possible coadjuvants in the control of type-2 diabetes and weight management following a chronologically planned snacking offer (morning and afternoon).

**Methods:**

The current trial followed a single group, sequential, longitudinal design, with two consecutive periods of 4 weeks each. A total of 17 volunteers participated in the study. The first period was a free living period, with volunteers' habitual *ad libitum *dietary pattern, while the second period was a free-living period with structured meal replacements at breakfast, morning snack and afternoon snack, which were exchanged by specific products with moderately high protein content and controlled low glycemic index, following a scheduled temporal consumption. Blood extractions were performed at the beginning and at the end of each period (free-living and intervention). Parameters analysed were: fasting glucose, insulin, glycosylated hemoglobin, total-, HDL- and LDL-cholesterol, triglyceride, C - reactive protein and Homocysteine concentrations. Postprandial glucose and insulin were also measured. Anthropometrical parameters were monitored each 2 weeks during the whole study.

**Results:**

A modest but significant (p = 0.002) reduction on body weight (1 kg) was observed during the intervention period, mainly due to the fat mass loss (0.8 kg, p = 0.02). This weight reduction was observed without apparently associated changes in total energy intake. None of the biochemical biomarkers measured was altered throughout the whole study.

**Conclusions:**

Small changes in the habitual dietary recommendations in type-2 diabetes patients by the inclusion of specific low-glycemic, moderately high-protein products in breakfast, morning and afternoon snacks may promote body weight and fat-mass loss, without apparently altering biochemical parameters and cardiovascular risk-related factors.

**Trial Registration:**

Trial registered at clinicaltrials.gov NCT01264523.

## Introduction

Type-2 diabetes prevalence in adults has grown in the last years in many societies, accompanying the high incidence of obesity-related and other cardiovascular risk factors [1]. Indeed, obesity is the most relevant overnutrition disease worldwide, being more dramatic than a self-esteem problem or an aesthetic issue, since it is associated to different metabolic disorders such as coronary diseases, hypertension, certain tumors, dislipidemia, biliary disorders, immunodeficiencies and insulin resistance [2].

Different studies have shown the efficacy of low-fat diets on weight reduction, which has been associated to an improvement in overweight-related chronic pathological conditions [3,4]. However, the concomitant high carbohydrate content in some weight-loss diets may imply the intake of foods with an elevated glycemic index, which leads to alterations in appetite directly associated to weight regain [5]. Furthermore, these commonly sugar-rich diets may exert negative effects on lipid profile [6] and insulin resistance [7]. In addition, there is increasing evidence about the key role of the quantity and quality of carbohydrates in diets, on the risk of developing chronic diseases [7-11]. Moreover, diets with a high glycemic load have been related to a higher risk of developing diabetes [6], while studies carried out with foods and diets of low glycemic load and high protein content have been associated with a reduction on LDL-cholesterol and triglyceride concentrations, as well as a lower postprandial glucose curve and an increase in HDL-cholesterol levels [7-14].

A number of trials have also demonstrated that diets with a low glycemic load increase satiety, reduce energy intake and produce a raised thermogenic effect [15,16], favoring weight loss and subsequently reducing cardiovascular disease and diabetes manifestations, although this apparently higher efficacy of diets with this macronutrient approach remains as a debate issue [17].

Additional investigations have shown that meals and diets with high protein content may be effective to combat the most important metabolic alterations observed in diabetic patients [7,12,18-20]. Additionally, some studies have demonstrated beneficial effects associated to dietary interventions based on specific breakfast patterns [12,19,20].

The present study aimed at researching the potential functionality of a series of low glycemic index products with a moderately high protein content, as possible coadjuvants in the control of type 2-diabetes and weight management within a nutritionally balanced dietary pattern, following a chronologically planned snacking offer (morning and afternoon), as well as the influence on biomarkers of the metabolic syndrome and cardiovascular risk manifestations.

## Methods

The study protocol was approved by the Clinical Research Ethics Committee of the University of Navarra (reference: 078/2009), and was registered at clinicaltrials.gov (identifier: NCT01264523). The full trial protocol can be accessed in Clinical Trials website (clinicaltrials.gov) by introducing the identifier. Recruitment took part during February - March 2010 and the study finished in June 2010. Written informed consent was given by all the volunteers participating in the intervention.

Taking into account the design of the present study as a single group longitudinal intervention, the authors have tried to fulfill the CONSORT 2010 guidelines [21], except for those points where it was considered non-applicable, such as blinding.

### Study design

The present trial was developed at the facilities of the University of Navarra with 17 participants, and followed a longitudinal design, with two consecutive periods of 4 weeks each. The first period, control phase, was a free living diet and status, where the patients followed their habitual *ad libitum *dietary pattern (following the recommendations of their physician), while the second period consisted on an intervention phase with structured meal replacements, where the volunteers' habitual breakfast, morning snack and afternoon snack, were exchanged by specific products, provided by the researchers, with a moderately high protein content and a controlled low glycemic index, following a scheduled temporal consumption. Therefore, the measurements and evaluation in the first period are considered as control values in relation to the intervention phase.

Blood extractions were performed at recruitment, if the volunteers didn't have a recent blood analysis (during the last three months), and on week 0 (beginning of the free living period), week 4 (end of free-living period and beginning of the intervention period) and week 8 (end of the intervention period). Anthropometrical parameters were also measured on weeks 0, 2, 4, 6 and 8 of the trial.

The primary outcomes of the current intervention were body-weight and fat mass, while glucose and lipid metabolism, as well as selected cardiovascular risk biomarkers were established as secondary outcomes.

Volunteers were asked to maintain their physical activity throughout the whole intervention. An important increase/decrease of physical activity compared to the baseline estimation was considered an exclusion criteria. Physical activity was estimated with a 24 h recall at the beginning and the end of the nutritional intervention.

### Products assayed

The products employed for the intervention trial (Enerzona^©^) were supplied by Equipe Enervit. These products are manufactured following a characteristic 40-30-30 energy distribution, with 40% of energy provided by proteins, 30% energy provided by carbohydrates and 30% energy provided by lipids. Additionally, all products are of low glycemic index (under 55 units). The variety of products assayed (Table 1), which volunteers had to consume all of them, with alternate options for each day, consisted on bars (27 g) with different flavors (orange, coconut, vanilla, cocoa and yoghourt), milk shakes (50 g) to dissolve in 150 mL semi-skimmed milk (chocolate, strawberry-yoghourt and cappuccino flavors), salted snacks (25 g packs, black olives and mediterranean style), biscuits (50 g/8 units, coconut, cocoa and oat) and minirock snacks (25 g/pack, soy and chocolate chips).

**Table 1:** Substitution guidelines during the intervention period of the volunteers' habitual breakfast, morning and afternoon snack.

Breakfast (choose alternatively one option each day)	
**Females**	**Males**

• 3 bars	• 4 bars
or	or

• 2 bars plus a glass (250 mL) of semi-skimmed milk	• 3 bars plus a glass (250 mL) of semi-skimmed milk
or	or

• 1 milk shake (disolved in 150 mL semi-skimmed milk)	• 1 milk shake (disolved in 150 mL semi-skimmed milk) + 4 biscuits
or	or

• 1 glass (250 mL) of semi-skimmed milk plus 8 biscuits	• 1 glass (250 mL) of semi-skimmed milk plus 8 biscuits plus 1 bar

**Morning and afternoon snack (choose alternatively one option each day)**	

**Females**	**Males**

• 1 bar	• 1 bar
or	or

• 1 salted snack (25 g)	• 1 salted snack (25 g)
or	or

• 1 Minirock (25 g - soy and chocolate snacks) pack	• 1 Minirock (25 g - soy and chocolate snacks) pack

### Study volunteers

Subjects participating in the nutritional intervention had to fulfill the following inclusion criteria: to be type-2 diabetes diagnosed patients, aged between 45 and 75 years old, following the dietary recommendations prescribed by their primary care physician, and eventually treated only with metformin (stable dosage during at least three months).

Exclusion criteria, which were controlled by a specifically trained physician, were to have a BMI under 22 or over 35 kg/m^2^, to follow a pharmacological treatment with other drugs but metformin, or being already insulin-dependent, to have other concomitant pharmacological treatments for weight loss, hormonal substitutive therapy, altered thyroid function, etc. without an stable dosage (at least three months prior the beginning of the study).

Additional exclusion criteria were to suffer from complications due to type 2 diabetes (microangiopathy, polyneuropathy, cardiopathy, hepatic and renal impairments, etc) or having a recent (within the three months prior to the beginning of the study) uncontrolled diagnostic of hypercholesterolemia and/or hypertryglyceridemia.

### Sample size calculation

Assuming a maximum loss of 0.6 kg/week, and expecting a total weight loss during the intervention period of 2.4 ± 2.5 kg compared to the free-living period, for an α value of 0.05 (5%) and an statistical power of 80%, the number of participants needed was estimated at 13 volunteers. Assuming an expected 20% drop-out during the trial, the minimum sample size required was established at 16 volunteers.

### Anthropometrical measures

Body weight, and body composition status were measured by a bioimpedance equipment (Tanita SC-330, Tanita corp, Japan), waist and hip circumferences were measured with a commercial measure tap following validated protocols [22]. Measures were taken with the participants in a fasting state of at least 8 hours.

### Glucose metabolism determinations

Fasting glycosylated hemoglobin levels were assessed at the Clinica Universidad de Navarra (Pamplona, Spain), by a high pressure liquid chromatography (HPLC) methodology [23]. Serum glucose was measured in an autoanalyser Pentra C-200 (HORIBA ABX, Madrid, Spain) and insulin concentrations were determined by an enzyme-linked immunosorbent assay (ELISA) kit (Mercodia, Uppsala, Sweden) in a Triturus autoanalyser (Grifols SA, Barcelona, Spain). Insulin resistance was estimated by the Homeostasis Model Assessment Index (HOMA-IR), which was calculated as stated in the following formula [24]: HOMA-IR = [glucose (mmol/L) × insulin (µU/ml)]/22, 5

Postprandial serum glucose and insulin levels were also measured at 30, 60 and 120 minutes after the consumption of both the habitual breakfast and the test breakfast with 40-30-30 products at weeks 4 and 8, respectively.

### Lipid metabolism variables

Total cholesterol, HDL-cholesterol and triglyceride serum concentrations were measured in an autoanalyser Pentra C-200 (HORIBA ABX, Madrid, Spain). LDL-cholesterol levels were calculated following the Friedewald formula [25].

### Inflammatory markers

C - reactive protein concentrations were analysed by an ELISA assay (Inmunodiagnostics, MA, USA) in a Triturus autoanalyser (Grifols SA, Barcelona, Spain). Homocysteine was determined in an autoanalyser Pentra C-200 (HORIBA ABX, Madrid, Spain).

### Dietary intake and satiety assessment

During the free-living period and during the nutritional intervention period, participants filled two 72 h dietary records, in which they must declare all the foods and quantities they had eaten in each period [26]. These questionnaires were further analysed with the DIAL software (Alce Ingenieria, Madrid, Spain).

Satiety was measured through self-reported questionnaires previously validated [27], based on a Visual Analogue Scale (VAS). Volunteers filled a total of four questionnaires during the postprandial glucose curves (before, having breakfast, 30, 60 and 120 minutes after having breakfast) at weeks 4 and 8.

### Statistics

The differences on variables between the beginning and the end of each period were analysed by a paired t-test, while the analysis of differences between both periods (free-living *vs*. intervention) was performed through an independent measures t-test. Postprandial glucose and insulin concentrations were analysed through a repeated measures ANOVA. Values of p < 0.05 were considered as statistically significant. All the statistical analysis were performed with the SPSS 15.1 software for Windows (SPSS Inc, Chicago, USA).

## Results

### Adherence to the nutritional intervention

Fifty-two subjects suffering from type-2 diabetes demonstrated an interest in participating on the study. Once at the Metabolic Unit, staff explained to them the complete protocol of the present nutritional intervention, 12 subjects declined the invitation to participate, and another 23 patients were excluded for not fulfilling the inclusion criteria: 11 were on another pharmacological treatment but not metformin, 6 subjects had a recent diabetes diagnostic and treatment was not yet established and 5 subjects were not within the requested age range, while another subject exceeded BMI criteria.

Finally, 17 selected volunteers started the study, from which 2 subjects withdrew before the final visit, whose baseline anthropometrical characteristics of the participants are reported (Table 2).

**Table 2:** Anthropometrical baseline characteristics of the subjects who completed the study (n = 15).

VARIABLE	MEAN	SD
Weight (kg)	82.5	12.7
BMI (kg/m^2^)	28.6	4.3
Waist circumference (cm)	102.0	10.7
Hip circumference (cm)	102.7	9.8
Fat mass (%)	29.5	8.1
Fat-free mass (kg)	58.4	6.9
Water (%)	50.7	2.9

There were no differences in anthropometrical features at the beginning of the second phase of the study compared to the values shown.

### Changes in anthropometrical parameters

**Figure 1:**
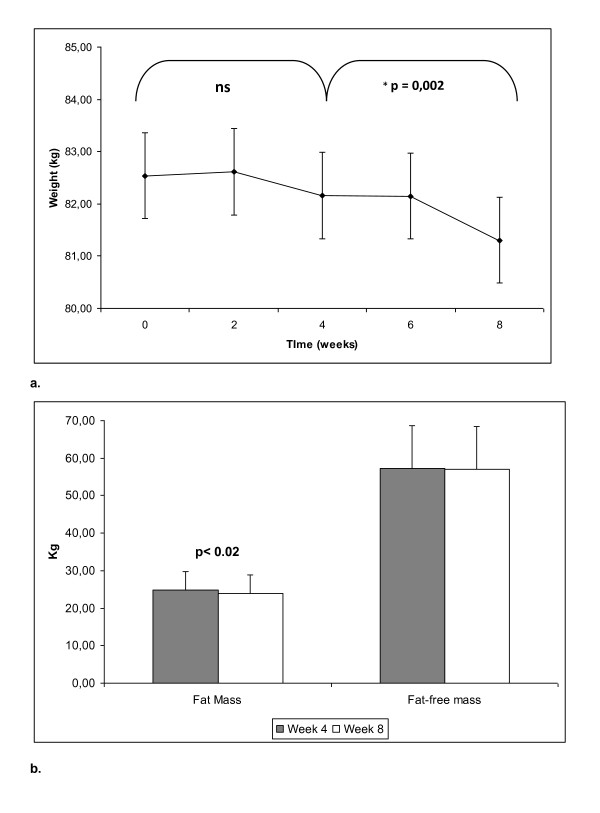
**Anthropometrical changes during the study**. The free living period corresponds to week 0 - week 4, while the nutritional intervention with 40-30-30 products corresponds to weeks 4-8. 1a: Body weight evolution. 1b: Fat mass Vs Fat-free mass.

Initial BMI on volunteers was of 28.6 kg/m^2^. Body weight remained unchanged during the free-living period (from week 0 to week 4), while a statistically significant decrease of about 1 kg was detected (Figure 1a) during the intervention period (week 4-week 8). Interestingly this change was observed without apparently associated changes in total energy intake (Table 3). In fact, energy intake was slightly higher in the intervention period (ns). This weight reduction was associated to a marked (p < 0.02) fat mass loss (0.8 kg), without statistically significant changes in fat-free mass (Figure 1b).

**Table 3:** Changes in macronutrient intake between the free-living and the intervention period

	FREE-LIVING PERIOD (WEEK 0 TO 4)						INTERVENTION PERIOD (WEEK 4 TO 8)					
	**Daily intake**	**Breakfast**	**Morning snack**	**Lunch**	**Afternoon snack**	**Dinner**	**Daily intake**	**Breakfast**	**Morning snack**	**Lunch**	**Afternoon snack**	**Dinner**

Energy (kcal)	1710 ± 354	300 ± 168	93 ± 86	713 ± 115	110 ± 86	493 ± 223	1815 ± 335	346 ± 59	111 ± 54	722 ± 154	111 ± 66	524 ± 238
Carbohydrates (g)	152 ± 52 (36)	39 ± 24 (52)	12 ± 11 (52)	51 ± 21 (29)	12 ± 11 (44)	38 ± 23 (31)	145 ± 48 (32)	34 ± 6 (39)	10 ± 4 (36)	53 ± 29 (30)	10 ± 6 (36)	38 ± 18 (29)
- Sugars (g)	73 ± 25	23 ± 17	8 ± 8	17 ± 7	7 ± 7	18 ± 8	72 ± 16	26 ± 6	7 ± 4	15 ± 7	7 ± 5	15 ± 9
Glycemic Index		51 ± 10	43 ± 22		37 ± 21			58 ± 9	40 ± 10		40 ± 10	
Fiber (g)	20 ± 8	4 ± 3	1 ± 1	9 ± 6	1 ± 1	5 ± 2	22 ± 7	4 ± 1	1 ± 1	10 ± 5	1 ± 1	5 ± 2
Proteins (g)	78 ± 16* (18)	11 ± 6* (15)	4 ± 5* (17)	37 ± 7 (21)	4 ± 4 (15)	21 ± 13 (17)	100 ± 15* (22)	25 ± 5* (29)	7 ± 3* (25)	36 ± 9 (20)	7 ± 5 (25)	24 ± 11 (18)
Lipids (g)	74 ± 13 (39)	10 ± 6 (30)	2 ± 2 (19)	33 ± 6 (42)	4 ± 3 (33)	24 ± 12 (44)	79 ± 15 (39)	12 ± 2 (31)	4 ± 1 (32)	35 ± 9 (44)	4 ± 2 (32)	26 ± 11 (45)

Values are Mean ± SD. Values within parentheses concern % of energy supplied by the respective macronutrients

* Significant differences (p < 0.05) between the free-living period and the intervention period

### Food intake and satiety assessment

Total energy intake did not greatly differ between the free-living and the nutritional intervention period (table 3), while protein intake significantly increased during the dietary snacking substitution with 40-30-30 products. Caloric profile in breakfast, morning and afternoon snack switched from a typical distribution around 15-55-30 (% protein-% carbohydrate-% lipid of the total energy intake) in the free-living period to the 40-30-30, during the intervention period. Glycemic index was also evaluated in the breakfast, morning snack and afternoon snack, and no significant differences were observed between both periods (table 3), despite that a global trend to increase was found as a consequence of 40-30-30 products consumption. No changes were observed in relation to physical activity during the intervention period (data not shown).

**Figure 2:**
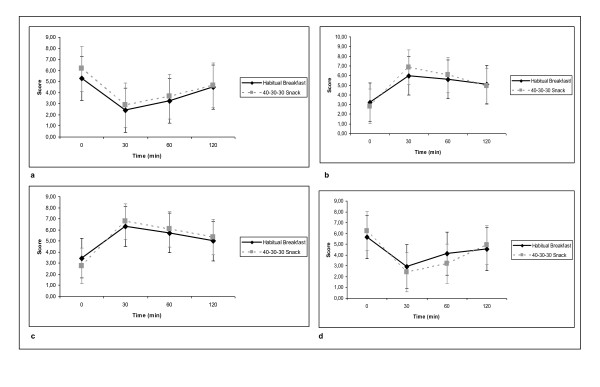
**VAS postprandial questionnaire results**. 2(a). Hunger score; 2(b). Satiety score; 2(c). Satisfaction score; 2(d) Intake score.

The analysis of the VAS questionnaire during the postprandial glucose curve, revealed no differences on hunger, satiety, satisfaction, intake and thirst (not shown), between the habitual breakfast or the 40-30-30 breakfast (Figure 2), while the values obtained during the 120 minutes postprandial state were as expected.

### Changes in biochemical biomarkers

**Figure 3:**
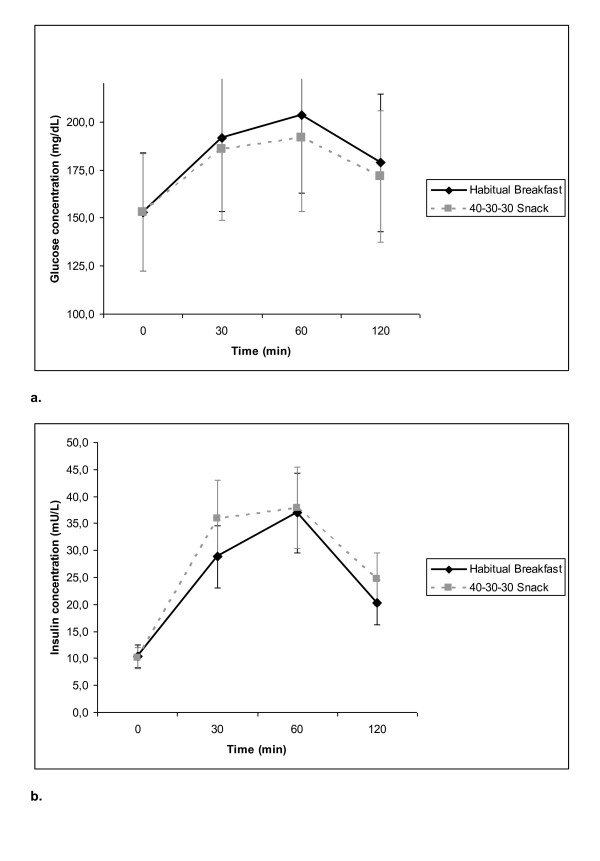
**Postprandial glucose and insulin curves either with the habitual breakfast and the 40-30-30 snacks, which were not significantly different**. 3a: Postprandial glucose curve. 3b: Postprandial insulin curve.

Blood analysis did not evidence statistically significant changes in any biochemical measurement during the whole study (table 4). Equally, postprandial glucose and insulin responses were similar in both periods (Figure 3).

**Table 4:** Changes in biochemical determinations between the beginning and the end of the study.

	Baseline	Final	p
Glucose (mg/dL)	159.2 ± 62.2	156.7 ± 59.4	ns
Insulin (mU/L)	10.2 ± 5.1	10.0 ± 4.9	ns
HOMA-IR	3.65 ± 2.00	3.43 ± 1.84	ns
Glycosylated Hemoglobin (%)	7.0 ± 1.3	7.2 ± 1.5	ns
Total cholesterol (mg/dL)	177.0 ± 37.4	176.9 ± 32.8	ns
HDL-cholesterol (mg/dL)	42.7 ± 9.0	42.5 ± 8.1	ns
LDL-cholesterol (mg/dL)	92.0 ± 37.2	102.9 ± 25.7	ns
Tryglycerides (mg/dL)	178.5 ± 103.6	157.7 ± 92.8	ns
AST (UI/L)	31.8 ± 22.2	26.9 ± 16.1	ns
ALT (UI/L)	36.6 ± 23.5	31.6 ± 19.5	ns
Uric acid (mg/dL)	5.8 ± 1.3	6.1 ± 1.5	ns
Homocysteine (μmol/L)	22.7 ± 4.4	23.3 ± 4.4	ns
C-reactive protein (mg/L)	13.6 ± 15.1	9.8 ± 10.7	ns

Values are Mean ± SD. No significant differences were observed.

## Discussion

The present trial reports the benefits of including within the habitual diet, different moderately high protein products (40% Carbohydrates-30% protein-30% lipids) for weight management, following a chronologically scheduled pattern. Thus it has been demonstrated that, within a free-living diet without dietary or energy restrictions, the substitution of a single meal by products with higher protein content resulted in a weight loss, due mainly to a fat mass reduction. In this sense, the results obtained with this work are in accordance with data previously reported [26,28-31].

*Ad libitum *diets with high protein intake have been considered as useful approaches for effective weight loss and later maintenance [11,28,29]. Indeed, some long-term studies with no calorie restriction and programmed macronutrient distribution have resulted in a more effective weight loss and maintenance than conventional macronutrient distribution energy-restricted diets [11,30,31]. In a recent study, an isocaloric diet with a moderately high content in protein led to a body weight and fat mass reduction after 10 weeks [26], without affecting fat-free mass and maintaining glucose and lipid profile, which matches with the outcomes of the current intervention.

The short duration of the present trial together with a total modest increase of the total protein intake (from 18 to 22% of caloric intake) may have played a role on the slight, although statistically significant, weight and fat mass reduction (approximately 1 kg). However, a period of 4 weeks is a good predictor of body fat changes with a dietary approach [32,33]. Indeed, when analyzing meals separately, the total protein content in breakfast, morning and afternoon snack has been increased in a range from 50 to 95% during the intervention compared to the free-living period, which seemed to be enough to produce a body weight reduction without caloric restriction, and gives support about the importance on timing of energy consumption, meal frequency and nutrient quality intake for weight maintenance [3,4,8,11,12,34].

The combination of protein content and glycemic index in a diet could be determinant on body composition changes [11,35]. In a recent study in Europe [11], it has been demonstrated the efficacy of high-protein, low-glycemic index diets on adults for weight maintenance [11]. In this same study, it was suggested that the isolated effect of protein content or glycemic index in *ad libitum* diets did not influence body weight in children, while the conjunction of these two dietary factors has been shown as protective against obesity [35]. Likewise, the current dietary intervention achieved an increase in protein intake together with a low glycemic index in the substituted meals, which may explain the observed improvement in body composition as assessed by bioimpedance measurements, a method which has been recently validated in our research group using Dual X-Ray Absorptiometry (DXA) as gold standard reference [22].

For the first time to our knowledge, it has been demonstrated that the inclusion of some specific meals in the habitual diet with high-protein low-glycemic index products may be sufficient for weight management, preserving lean mass and helping to decrease fat mass in type-2 diabetes patients, compared to a control period of free-living diet. These effects should be ascribed to the protein induced rise in thermogenesis [7,11,26,36,37], or even to the increased satiety consequence of high protein ingestion [38,39], although the assessed satiety scores have not reflected this effect in the current study.

In relation to the lack of changes concerning glucose and insulin levels as well as the insulin resistance index, it may be due to the short intervention period, although previous studies with similar or even shorter periods have shown clinically relevant effects with respect to an improvement on insulin sensitivity [9].

Furthermore, contrasting with other nutritional intervention studies, our results did not show differences between the free-living and the intervention periods on lipid metabolism. Several studies have evidenced that exchanging protein for fat improves lipid-related cardiovascular risk profile [36,40]. However, most of these studies used energy-restricted, low-fat, high-protein diets [37,40-43]. Indeed, all the foods included in these hypocaloric diets were low fat products, decreasing also saturated fat intake, which could be one of the main factors involved in the reduction of total and LDL cholesterol. On the other hand and in agreement with our findings, several studies comparing high protein diets have not observed changes in lipid profile, concretely on total and LDL cholesterol [7,37,42]. However, a recent study by Morenga and co-workers [36] found that an ad libitum diet relatively high in protein improved total cholesterol and low-density lipoprotein cholesterol in comparison with standard dietary advice. In this case, the fiber content of the diet was also relatively high (>35 g per day), which could counteract the high fat consumption in this group [36]. It is important to point out that, in the present study, the driven substitution of specific single meals by 40-30-30 products, led to a modest increase in the total dietary daily protein content (22%), which is lower than the quantities routinely used in high protein intervention programmes [36].

High protein diets have been also related with reductions in triglyceride levels [36,37,44,45]. Comparing the duration of the present nutritional intervention period to other nutritional programmes, this one may have been relatively short to achieve significant changes in triglyceride levels. Indeed, triglyceride levels tended to be lower at the end of the nutritional intervention period in spite of not reaching statistical significance. Moreover, the effect on triglyceride levels has not always been observed in longer dietary interventions with a moderately high-protein content [7,46]

A limitation encountered for the implementation of the current nutritional intervention has been the low sample size and the limited duration of the intervention, which can not permit us to generalize the outcomes obtained without further research. However, it is generally assumed that finding statistical significance with a small population is more difficult than when having a higher sample size. This outcome usually indicates that there is a real difference between the experimental periods. In any case, a type II error can not be discarded [47]. Furthermore, as the study was designed as a longitudinal intervention with two consecutive periods, the first period is really a control, where the researchers only performed observational follow-up work. This approach has been already successfully employed and published elsewhere [48-51].

The overall results of this study may have been partly affected by the fact that the participants in the nutritional intervention were type-2 diabetes patients with initially controlled dietary treatment. In fact, the glycemic indexes in the breakfast, morning and afternoon snack during the free-living period were relatively low at baseline (< 55 units), and similar to the glycemic load reached with the products assayed. Thus, in another trial [38], the consumption of a low glycemic index breakfast during 21 days, compared to a high glycemic breakfast, led to a significant reduction on fasting glucose levels without affecting other biochemical biomarkers in obese subjects. In addition, a benefit on satiety was also reported, as it increased with the low glycemic index meal [38], which was not seen in the current trial. These observations suggest that in this population, the moment/time of consumption may be relevant in interpreting the results.

Moreover, when comparing two hypocaloric diets differing in the glycemic index, beneficial additional effects were found after weight loss (-5.3% *vs. -*7.5% change with the high- or low-glycemic diet, respectively) as well as in total- and LDL-cholesterol concentrations, where the decrease was 4-fold higher in the low glycemic index diet [52].

The reduction of glycemic index in a specific meal [38] or a diet [39] has also been associated to an increase in the satiety and a reduction on the voluntary food intake during the postprandial state. Indeed, the voluntary food intake may be an 80% higher after the consumption of a high glycemic *vs*. a low glycemic index meal [39]. In this context, it is also in agreement with previous studies the similarity of the satiety scores observed between the free-living period and the intervention period in the present trial, as glycemic index remained unchanged between both periods.

The present results together with those from others [19,34,38] indicate the evident benefits of nutritional interventions on selected meals, giving an increasing importance to chrononutrition and meal frequency intake. Therefore, this is a good example of translational research carried out in a limited number of volunteers.

## Conclusions

Summing up, the present trial evidences that small changes in the habitual dietary recommendations in type-2 diabetes patients by the inclusion of specific low-glycemic, moderately high-protein products in breakfast, morning and afternoon snacks may promote body weight and fat-mass loss, without apparently altering biochemical parameters and cardiovascular risk-related factors. The importance of these findings are related to the novelty of demonstrating that increasing protein content of selected meals offered in specific moments (breakfast, morning and afternoon snack) leads to an improvement in body composition, and it is also important to highlight the effect of regulating meal frequency and timing as a basis for future research concerning chrononutrition.

## Competing interests

The authors declare that they have no competing interests.

## Authors' contributions

SNC participated in the design of the study, development of the trial, outcomes measurements, data analysis and drafted the final manuscript.

IA participated in the design of the study, outcomes measurements and drafted the final manuscript.

MAZ participated in the design of the study, data analysis and helped to draft the final manuscript.

JAM conceived the study, participated on its design and coordination and performed a critical review of the final manuscript. He also managed the funding to carry out the intervention.

All authors read and approved the final manuscript.
